# Children’s Nature Use and Related Constraints: Nationwide Parental Surveys from Norway in 2013 and 2023

**DOI:** 10.3390/ijerph22071067

**Published:** 2025-07-03

**Authors:** Vegard Gundersen, Zander Venter, Odd Inge Vistad, Berit Junker-Köhler, Line Camilla Wold

**Affiliations:** 1 Norwegian Institute for Nature Research, NO-2624 Lillehammer, Norway; odd.inge.vistad@nina.no (O.I.V.); berit.kohler@nina.no (B.J.-K.); line.wold@nina.no (L.C.W.); 2 Norwegian Institute for Nature Research, NO-0855 Oslo, Norway; zander.venter@nina.no

**Keywords:** neighborhood, nature contact, outdoor play, spontaneous play, urban nature, childhood, natural environment, urban forest

## Abstract

A growing number of research studies show that children spend less time in natural environments, which may have detrimental effects on children’s mental and physical health. This study explores changes in children’s (6–12 years) use of nearby nature and constraints on playing in nature between 2013 and 2023. We apply an ecological approach, including individual, social, and structural constraints on outdoor play. The study is based on national surveys of parents reporting child play behavior for eight activity categories and nineteen categories of constraints/motivation. Findings reveal a decreasing tendency for time spent on all activity categories and increasing constraints for 17 of 19 categories during the study period. Our ecological approach reveals that there is less time for children’s nature use in contemporary society, and activities are more common in built areas than in nature. The survey identifies some important socio-cultural differences regarding gender, age, and residential setting. In future research, the focus should be on how reduced connection to nature affects children’s mental and physical health, and beyond this, how it affects the understanding of and care for nature among future generations.

## 1. Introduction

A growing number of research studies show that children spend less time in natural environments (e.g., [1,2]). This happens despite increasing knowledge about the importance of natural environments for children’s physical and mental health, well-being, and development (e.g., [3,4,5,6]). Nature experiences and contact have similar relaxing and restorative effects for children as for adults [7]. Playing is important in children’s individual development: social, linguistic, physical, emotional, and cognitive [8,9], as well as the development of morale, creativity, and problem-solving skills [3,10,11]. Playing is also cultural, where access to different types of play, including independent play in natural environments and on playgrounds, helps to socialize children as participants in society [5,12,13]. Because play and nature contact are considered important for children, children’s right to play is also enshrined in the UN Convention on the Rights of the Child [14].

Children’s engagement with nature not only depends on structural constraints, such as physical access and the quality of the natural environments, but it is closely interwoven with all aspects of their everyday life [15,16]. Today’s children are more engaged in organized activities which compete with their need for leisure time [17,18]. Changing lifestyles, with more focus on screen activities and coping with the pandemic and safety concerns within families, have caused a shift toward spending free time indoors rather than outdoors in natural environments [2,19]. In a situation where children spend more time indoors, especially during the COVID-19 lockdown (e.g., [20,21]), there is a lower likelihood of meeting friends outdoors spontaneously, as was common in the past [17]. But it is not only about structural and social constraints on children’s engagement with nature. It is also about parents’ and children’s (lack of) individual and group dispositions, motivations, and preferences for playing outdoors [8,22].

There is also a question of social justice in understanding constraints on access, and in developing different arenas that improve the possibilities for nature engagement across all kinds of family situations [23,24]. Despite growing international research on constraints on children’s nature engagement (e.g., [15,24,25,26,27]), there is a need for more contextual knowledge about the constraints in different socio-cultural and environmental settings, and a better understanding of family and everyday life situations that increase or decrease the possibilities for children’s engagement with nature.

We take a contextual ecological approach, including political, social, cultural, and economic aspects, to study constraints on nature play [18,28]. In this paper, we consider a simplified nature continuum that spans different levels of human intervention and biophysical components—from spaces such as playgrounds at the developed end, to more natural areas like forests at the other end. All environments along this continuum contain varying amounts of natural or green space elements. Taking “green” Norway as a case is highly relevant for investigating the multifaceted character of the topic, and of international interest due to similarities in contemporary childhood across Western countries [29]. Norway offers excellent opportunities for children to engage with nature, both because of a high proportion of natural areas in residential settings, free access to all nature regardless of land ownership, and a strong cultural tradition of being out in nature, exercising “friluftsliv” [30]. In all, 97% of parents of children aged 6 to 12 years reported that their child had good access to nature within walking and cycling distance from home [31]. Being out in nature during childhood is strongly valued among Norwegians and has been associated with rough play intended to make children robust, rational, and independent. However, society is changing, and children’s engagement with nature no longer seems to be an integral part of everyday life, as forests and fields are used much less by children compared to built areas [31], and their activities are to a large extent supervised by adults [10,30]. As much as 98% of the Norwegian children participate in outdoor recreation with their families at least once a year; however, the frequency of visits has decreased by 10% between the years of 2007 and 2020 [32], which corresponds to a similar downward trend in other Western societies (e.g., [33]). In the context of “green” Norway, it is interesting to study children’s engagement with nature and how this has changed over time. We collected national questionnaire parental data in 2013 and 2023 that included children aged 6 to 12 years. The following research questions guide our research:

- Which outdoor activities are Norwegian children engaged in, how often, and what are the main changes?
- What are the main constraints on children’s nature engagement, and what are the main changes?
- What kinds of demographic and social variables may explain the observed pattern of constraints?

## 2. Constraints on Children’s Use of Nearby Nature

### 2.1. An Ecological Model for Studying Constraints for Outdoor Play

The diversity of literature on why children do not play in nature—including studies from Norway [18]—calls for a holistic theoretical perspective when studying constraints on play. Such a perspective should include physical attributes of the environment, as well as individual and social aspects of children’s everyday lives in contemporary society. This broad life perspective is the basis for an article where the authors call for “an ecological approach to building active living societies”—and their motive is “to achieve population change in physical activity” [28]. This approach is relevant for children’s play and engagement with nature as well, and it indicates that the responsibility for children’s activities is primarily a social responsibility, not an individual one. Our paper examines what constrains children from using their neighborhood, and particularly the potential of the natural environment for active outdoor play in their leisure time. Increasing access to nature spaces and developing infrastructure for outdoor activities is insufficient to promote outdoor play. It is crucial to look at wider challenges linked to the social, economic, cultural, and legal aspects. Some have stated that one must build a lifestyle and a culture where being inside doing screen-based and passive activities becomes abnormal, and where children’s outdoor play is the norm [34].

**Figure 1:**
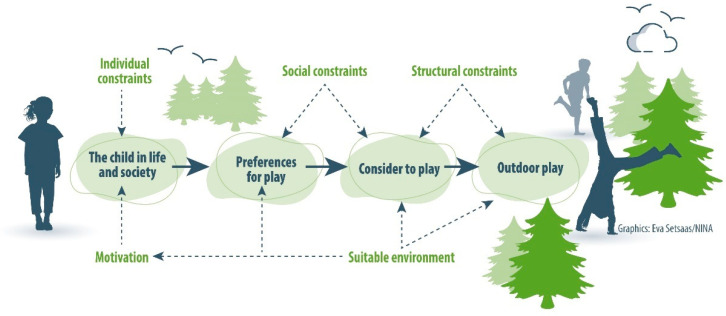
Illustration of the main elements (various constraints, motivation, and quality of the environment) influencing children’s actual outdoor play; see text for explanations. (Outlined from leisure literature [35,36]).

Constraints on outdoor play are complex [15,30], something which is confirmed in the ecological approach to active living [28]. We have chosen to use the term ‘constraints’, to signalize that it includes all aspects of “fixed conditions” (e.g., gender, ethnicity, disabilities, etc.) and also aspects that can be influenced and altered (e.g., lack of knowledge, time constraints, the social situation, the environment). However, the term ‘barrier’ (and hindrances) that is commonly used in outdoor play literature (e.g., [16]) is often more narrowly defined. The term ‘barrier’ is used to seek practical solutions to overcome individual barriers, especially with attention to the external physical environment and what creates a barrier between personal (play) preference and actual personal participation in play activity (e.g., [25]). The term ‘constraints‘ refers to a broader complexity and structure, ranging from personal mental constraints to unsafe or unsuitable outdoor environments (Figure 1). Knowledge about constraints must thus be placed in a larger context, since removing, for example, personal, or structural constraints has little effect if the parents or children do not have the motivation for outdoor play. The literature does not count “lack of desire to play” as a constraint, but rather as a lack of motivation (e.g., [35,36]). However, ‘motivation for outdoor play’ is also complex and dependent on many variables—both internal/personal and external/societal. The most important reason why many children do not play outdoors is likely a lack of motivation or interest in being outdoors, combined with a stronger preference for indoor screen activities [19,34].

In addition to having a motivation to play outdoors, the quality of the outdoor environment must be attractive and suitable in terms of recreational facilities and amenities, have high availability and accessibility, and be safe [27]. Some researchers use the term “physical affordance” to describe this phenomenon [36]: whether an object or a physical environment ‘invites’ a specific activity or not—for example, a natural environment that is like an “open book” of play possibilities, or a swing that invites you to swing [37]. In Figure 1, we have translated ‘physical affordance’ into ‘suitable environments’. Diversity in play facilities is a key factor in understanding the possibilities for play and social interaction for children in the neighborhood, including both play equipment and non-play features of natural elements [13]. A study of the behavior of small children during the redesign of their childcare outdoor play space (i.e., a developed, not a natural area), found that qualities like edges, levels, and inclines triggered the intensity and variety of children’s physical activity, and led to greater utilization of the space [38]. These three qualities are also typical characteristics of natural areas. They highlight the value of considering children’s own perspectives on the play environment and its design. Hence, they also draw attention to the influence of caregivers: children playing on their own may behave very differently from when they play under the supervision of educators or other adults [30].

Previous research has identified several individual, societal, and structural constraints that may restrict individuals from participating in outdoor nature activities (e.g., [26,35,36]). We use a three-step framework for studying constraints related to children’s nature contact, which divides constraints into three categories (Figure 1).

1. Individual, also called ‘intra-personal’ (especially in psychological terms), such as self-image, interests/preferences, age, stage of life, physical health, knowledge, disability, anxiety, fear, attitudes, and personal norms.
2. Social, called ‘inter-personal’, such as social circle, lack of play companions, family responsibilities, and a social network for outdoor play.
3. Structural, relating to both the private and external environment, such as the socio-cultural factors, economic factors, transportation, time constraints, physical access to play in areas, and the distance to and quality of outdoor spaces. Institutional constraints (e.g., fees, restrictions) are included in this category but are considered less important in Norway due to common rights of access.

### 2.2. Motivation and Individual/Intra-Personal Constraints

Lack of motivation is not considered a barrier (see Figure 1), but children’s interests/preferences for spending leisure time indoors—doing screen-based activities or organized sports activities—as well as adults’ preferences for other activities such as indoor play and doing schoolwork, constrain the possibilities for outdoor play [30]. Individual constraints are closely related to parents’ level of knowledge about the benefits, motivation, and hopes they hold for their children to play outdoors [39]. Adults’ norms and attitudes are crucial for motivating children towards independent outdoor mobility and play [29].

Internationally, outdoor play activities have become increasingly limited for many children due to excessive fear of risk among adults [30,40,41]. When children receive constant warnings from adults about ordinary activities such as climbing, swinging, jumping, or playing in the forest, adults may unintentionally transfer these fears to the children [18]. The literature emphasizes that fear of stranger danger, dangerous streets (violence, harassment, and drugs), and car traffic is common among parents [41,42]. Many educators and parents feel a personal responsibility to assess risks and supervise children. However, perceptions of risk are highly subject to cultural interpretation [12]. Compared to many other Western countries, Norwegian children’s independent play still appears to be an important part of childhood, as it is across the rest of Fennoscandia [29]. Most studies in Fennoscandia focus on safety and risk in preschool and kindergarten, rather than on the broader perspective of children’s leisure time play [18]. Some suggest that this reflects a child-rearing culture that values children experiencing challenging situations and learning through embodied experiences and risk mastery [30]. For example, Norwegian researchers emphasize the importance of independent outdoor play, which is often risky, chaotic, and noisy, but provides many benefits for the development of social, psychological, and physical skills [43,44]. However, a study from Sweden identified mobility restrictions by adults in urban settings due to concerns about traffic and stranger danger. Thus, the picture is more diverse in Fennoscandia today than in the past [10].

### 2.3. Social/Inter-Personal Constraints

Only a few decades ago, playing in the neighborhood without adult supervision was a very important social arena for children. Children met informally, and it was common to ring the doorbell and ask, “Come out and play”? after school [17]. When a child goes out in the neighborhood today, there are usually no other children around, and thus the motivation to go out to play is diminished. What is regarded as “normal afternoon behavior” for a child seems to have changed radically in recent decades. Today, children mostly meet friends in organized or institutional settings [45]. These changes are fundamental and apply to both urban and rural contexts [46]. Hence, it is up to the parents whether they bring their children out into nature or have the social networks to organize informal meetings or hikes for outdoor play. Both children’s and parents’ social networks have changed, and this constrains children’s outdoor play and play in natural settings [37].

### 2.4. Structural Constraints and Environmental Quality

A suitable environment is an important variable, including both accessibility to suitable areas and an inviting/attractive physical environment [27,37]. Loss of green spaces in urban areas—especially as part of new sustainable compact cities models—is a major concern for children’s opportunities to play outdoors in many Western countries [23]. Norway, as well as other Fennoscandian countries, such as Sweden and Finland [31,37], has particularly good opportunities for children’s outdoor play due to public access rights to uncultivated land, regardless of ownership. However, socio-cultural changes in society are influencing children’s actual use of outdoor spaces for playing in Norway, much like in other European countries [18,29]. For example, today, 93.4% of Norwegian preschool children attend kindergarten [32], and there has been a long-standing tradition of prioritizing independent play in nature in the education of preschool teachers in Norway (e.g., [42,47]). However, there are also clear indications that some kindergartens and lower secondary schools now have less play space, and that the quality of outdoor areas has changed—shifting from informal nature areas to developed spaces with built play installations [48].

Opportunities for play and accessibility to nature reflect how children and their parents are influenced by socio-environmental factors—for example, how the physical environment may restrict participation [37]. A common theme in international literature today is parental control over children’s leisure time [30,49]. Some researchers refer to this phenomenon as the “domesticated child” [12], where parents closely monitor their children and organize their leisure activities, meaning children rarely spend time outdoors on their own or without supervision [17]. In a Norwegian survey, 30% of parents reported that their children never go outdoors without them, and that their children spend more time outdoors with their adults/parents than without [18]. This underscores that children’s use of nature largely depends on their parents’ motivation to take them outside [37]. Additionally, parents are often busy with work and their own leisure activities, and a lack of time becomes another structural constraint [46].

Modern society has also seen a growing and persistent perception of risk (e.g., [50]), resulting in significant restrictions on outdoor play among today’s children compared to previous generations [51]. A challenging environment for the child may stimulate independent play, but adults must find a balance between encouraging risky play and ensuring safety [50,52]. This is especially relevant in institutional and educational settings like kindergarten, preschools, and primary schools, where adult supervision is common and children have less time for independent play [45]. Some researchers advocate that children’s opportunities to play should not be determined solely by adult concerns and risk assessments [42,44].

Another important constraint for children’s outdoor play is modern technology—especially their preference for staying inside with screen-based activities or digital games [20]. Children in Norway live in a prosperous society with widespread access to digital media. A national survey conducted for the Norwegian Media Authority in 2023 found that 93% of children aged 9 to 11 had access to a smartphone (up from 84% in 2014) [53]. Both the proportion of users and time spent on screens have increased over the last decade, and usage increases with age. A worldwide shift in media practices, including among children, has become evident. Additionally, the market for computer games has expanded significantly—particularly among girls—and many TV channels target children with programming immediately after school time [53]. The consequences, such as increased inactivity and child obesity, are complex and also influenced by other factors such as social class, personality, and individual interests [20]. Children’s time use should be seen as a complex and dynamic issue situated within specific cultural contexts [18]. Nonetheless, the shift in media practices has undoubtedly contributed to the declining appeal of outdoor spaces as attractive play environments for children.

About 63% of children and youth aged 6–19 in Norway participate in organized leisure activities, and when including sports activities, the percentage rises to about 80% [46]. At the same time, children are spending increasing amounts of time in childcare, schools, and after-school care [32]. There is a highly target-oriented, scheduled, and at times overscheduled everyday life for children in Norway—similar to what is observed in international studies (e.g., [2]).

## 3. Material and Methods

### 3.1. Target Population, Sampling Technique, and Sample

The survey was administered in 2013 and 2023 to a representative nationwide Norwegian panel through the polling company Kantar. Kantar panel participants are recruited through a variety of online and offline methods, with more than 50% recruited via direct personal contact by telephone. The panelists consent to participate in survey-based research when they register to join the panel. The survey was web-based, and respondents answered directly via mobile phone, tablet, or PC. To ensure geographical representativeness, we stratified at the county level and included 15 counties. We invited parents and other types of caregivers (hereafter referred to collectively as ‘parents’) with children aged 6–12 years to participate. Respondents were initially screened through a question asking whether they were a parent of children aged 6–12. Subsequently, 3168 and 433 respondents completed the questionnaire in 2013 and 2023, respectively, representing our target population.

Currently, there is no standardized method for calculating a response rate for this type of survey, but sample bias in relation to the target population is considered an important quality indicator [22,54,55]. We considered the sample of respondents to be representative of the target population with respect to the children’s gender, parents’ age, geographical distribution across six regions, and family structure (whether the child lived in one home or more than one home) among the population of Norwegian children aged 6–12 years [22].

To avoid over-representation of parents whose children were particularly engaged in outdoor recreation activities, respondents were initially informed that the survey concerned children’s leisure time in general. We consider the sample valid for including children who were currently engaged in nature-based activities, had previously participated, or had participated in such activities [22]. Respondents were asked to answer the questionnaire on behalf of their oldest child within the specified age range (6–12 years). This resulted in a slight over-representation of parents of older children (particularly those aged 10–12) compared to younger ones (particularly those aged 6–8). As is common in such national surveys of parents [31], the proportion of immigrant parents in the sample was lower (10.2% in 2023, not measured in 2013) than in the official national statistics for the Norwegian population (17%) [22].

### 3.2. Questionnaire and Measurement Methods

Key survey content was developed based on existing literature and by applying an ecological model that included the main settings for the children’s leisure time. The surveys included questions on the parents’ and children’s demographics and their activities in different outdoor settings, and—based on a literature review—we selected 19 statements addressing constraints on engaging in outdoor play and time spent in natural environments (Supplementary Tables S1 and S2). The selected items used in this secondary analysis are listed in Table 1.

Responses to nineteen statements of constraints were reported using a 5-point Likert-type scale, ranging from ‘completely disagree’ (score 1) to ‘completely agree’ (score 5) (Table 1). In addition, we asked the parents to answer key demographic, socio-economic, and social questions (Table 1). The definitions of these standard background variables relating to parents and children followed official definitions for statistics in Norway [32], and they have been used in several similar national surveys targeting children and adolescents (e.g., [31,46]).

### 3.3. Data Processing and Analyses

After data collection was complete, cleaned datasets (.csv files) were received from the polling company. Further data cleaning and verification were completed by us. The data were analyzed using IBM’s SPSS Statistics 27. We decided not to weigh the dataset, i.e., by age, because the data analyses were robust without weighting. Overall, descriptive statistics were calculated for means (standard deviation) and percentages for all variables and were presented for selected variables. The robust Welch’s *t*-test was used to assess differences in means of barriers for outdoor use between the two populations, 2013 and 2023. Selected demographic, socio-economic, and social variables were collapsed into two levels. Associations between constraints on nature play and demographic, socio-economic, and social variables were assessed by Pearson point-biserial correlation. Statistical significance was set at *p* < 0.01, and correlation coefficients r > 0.1 are highlighted [20]. Effect size is determined by the r value, and r < 0.1 indicates small effects, r = 0.1–0.3 medium effects, and r > 0.5 large effects. A value near 0 indicates a weak or no relationship. We highlighted results that are larger than small effects (r > 0.1, Supplementary Tables S1 and S2). Analyses were completed and checked by two of the authors.

To assess the internal consistency of the constraint items, Cronbach’s alpha was calculated. The results showed high reliability, with α = 0.856 for the 2023 sample (n = 433) and α = 0.890 for the 2013 sample (n = 3168). These values indicate strong internal consistency for the measurement scale and are in line with findings from previous studies in this field.

### 3.4. Ethics Approval

The data used in the analysis was collected in line with ethical standards. The respondents, who had given prior consent to participate in research, were drawn from a panel managed by a third-party company, Kantar. Kantar has strict requirements for privacy and data security and works in accordance with both the Norwegian Data Protection Authority’s guidelines and the provisions of the Personal Data Act. Collection and analysis of data were carried out with approval from the Norwegian Centre for Research Data (NSD). The data file provided to us did not include any identifiable personal information and thus the participants were fully anonymized.

## 4. Results

### 4.1. Changes in Children’s Outdoor Use in Different Neighborhood Settings

**Figure 2:**
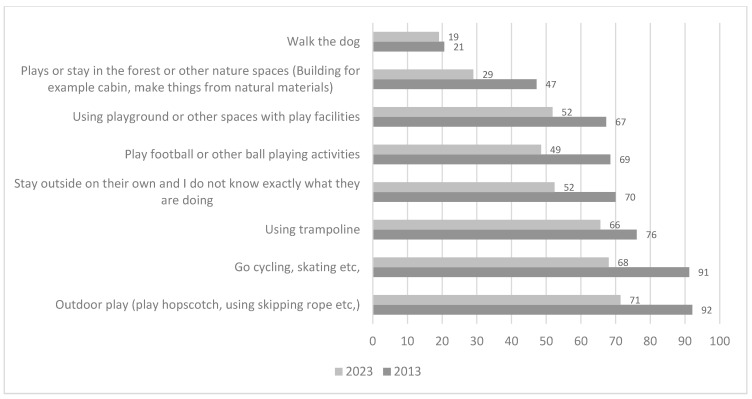
Percentages of frequent use (more often than weekly) for different leisure time activities in the neighborhood in 2013 and 2023.

Parents reported that their children engage in a variety of outdoor activities during summertime. The most frequent activities include outdoor play (e.g., hopscotch, skipping rope, meeting friends), cycling and skating, using a trampoline, playing football or other ball games, or going outdoors without parents knowing exactly what they are doing. However, the proportion of children doing these activities more often than on a weekly basis has declined between 2013 and 2023. In 2013, a total of 47% of children engaged in activities in forests more often than weekly; this figure declined to 29% in 2023. Apart from walking the dog, playing or staying in forests is the activity reported as least frequent on a weekly basis (Figure 2).

### 4.2. Changes in Parents’ Experiences of Constraints for Children and Youth to Be Outdoors in Natural Settings

The highest-rated constraints on being outdoors in natural settings for children are “The child is too busy in leisure time”, “The child prefers being indoors”, and “School homework takes too much time”. The lowest-rated constraints were “The child has poor motoric skill”, “The financial expense to reach attractive nature and green areas is too high”, and “I/we parents find it unsafe in nature and green areas” (Table 2). Concern about the traffic situation and excessive time spent on screens was also rated highly among the parents.

We observed significant differences between 2013 and 2023 for 17 of the 19 constraint variables, and all the significant constraint differences were rated higher in 2023 than in 2013. The only two exceptions to statements that did not show significant differences between 2013 and 2023 were “I/we parents are concerned about traffic” and “School homework takes too much time”. Robust tests for unequal sample size, including Welch’s *t*-test and the Brown-Forsythe test, produced consistent results. Regarding the five most important constraints (those with the highest means), four of them were the same in both the 2013 and 2023 samples (see Table 2).

### 4.3. Constraints on Being Outdoors Associated with Demographic, Socio-Economic, and Social Variables

Since there has been a negative trend in the outdoor use of children in the last ten years, and an increase in experienced constraints on outdoor use, it is interesting to dig deeper into the constraints. Details of the associations between 19 statements measuring constraints for children’s outdoor use of leisure time in 2013, and their allocations to demographic, socio-economic, and social variables are summarized (Supplementary Table S1). The following selected associations (r > 0.1, *p* < 0.01) are highlighted [20]. With regard to the statement that most parents evaluated as the highest hindrance, “The child is too busy in leisure time”, we identified three significant differences: the oldest child group (0.11), parents with the highest income (0.13), and those living in urban settings agreed most with this statement. For “The distance to nature and other green areas is too far”, those living in urban settings (0.15) agreed most. Parents with the youngest children group agreed most with the statement “I/we parents are concerned about traffic” (−0.15). Parents with the oldest children group agreed most with the following statements: “School homework takes too much time” (0.1), “The child prefers being indoors” (0.11), “The child uses so much time on data and other screens that to be outdoor is downgraded” (0.26), “The child does not want to play outdoors in nature” (0.17), and “The child lacks friends who want and have time to visit nature and green areas” (0.12). The statement “The child uses so much time on data and other screens that to be outdoor is downgraded” was agreed with most by parents of boys (−0.15). Those living in urban settings agreed most with the statement “I/we parents have a time schedule filled up with job, activities, sports, and other things and motivating the child to be outdoor is downgraded” (0.1). Single parents agreed most with “I/we parents lack a social network that could increase activity with the child outdoor” (−0.12). Women showed higher constraints than men for the following three statements: “Too high demand for equipment, cloths, shoes etc.” (−0.12), “The child uses so much time on data and other screens that being outdoors is downgraded” (−0.13), and “I/we parents find participation in sports and other leisure activities more important than motivating the child to be outdoor in nature and other green areas”. Parents of the highest age group agreed significantly more than the younger parents with the following statement: “The child uses so much time on data and other screens that to be outdoor is downgraded” (0.10). The variables parents’ education level (low/high), family structure (one home/two homes), country of origin, and number of children did not show any strong associations with the measured statements of constraints.

We identified 19 significant associations with the criterion (r > 0.1, *p* < 0.01) in the 2023 material (Supplementary Table S2). Parents of children in the oldest age group (9–12 years) were least concerned about the traffic situation (−0.23), but for this group the parents agreed most with these statements: “The child uses so much time on data and other screens that to be outdoor is downgraded” (0.18), “The child does not want to play outdoors in nature” (0.15), and “The child lacks friends who want and have time to visit nature and green areas” (0.14). Parents with the highest income agreed most with the following statement: “The child is too busy in leisure time” (0.15), while those with the lowest income agreed with the following three statements: “The child uses so much time on data and other screens that to be outdoor is downgraded” (−0.16), “The child does not want to play outdoors in nature” (−0.16), and “I/we parents lack a social network that could increase activity with the child outdoor” (−0.13). With regard to parents’ education (low/high), we identified that those with a low education level agreed more with the following statement: “School homework takes too much time”. We found that single parents reported stronger constraints for four items: poorly facilitated areas (−0.16), perceived lack of safety (−0.18), lack of a social network (−0.15), and prioritization of indoor activities (−0.12). Regarding parents’ age, the strongest constraints were reported by the youngest group: concern about traffic (−0.13), frequent bad weather (−0.14), poor motoric skills (−0.15), and perceived lack of safety (−0.13). For the child’s gender, rural vs. urban residency, family structure, country of origin, and number of children, no strong associations were identified.

## 5. Discussion and Conclusions

### 5.1. Comprehensive Decrease in Children’s Greenspace Use

We found that all eight categories for outdoor activities had decreased since 2013, and especially the category “play or stay in the forest or other nature spaces” has decreased from 47% who did this more often than on a weekly basis in 2013 to 29% in 2023. It is only the category “walk the dog” that has a lower frequency of use among children. Our results corroborate a well-established downward trend in play and time spent in nature for children and adolescents in Norway and elsewhere in the Western world (e.g., [30,33,49,56]), and debates about consequences for childhood (e.g., physical and mental health, friendship, social networks) and children’s further life opportunities [3,5,6,9]. Shifts in lifestyle patterns, characterized by increased engagement in screen-based activities and influenced by psychosocial responses to the COVID-19 pandemic and heightened concerns about familial safety, have contributed to a marked decline in outdoor recreation and a corresponding increase in time spent indoors, thereby reducing exposure to natural environments [2,19]. Explanations for this dramatically negative trend may be found in the constraints of not being outdoors for activities (see Section 5.3).

The data for children’s use of outdoor spaces shows that nearby nature spaces are not an integrated part of most children’s daily life, as seems to be the case for activities that typically take place in more developed outdoor spaces. This is in line with a study that identified a decrease in the number of children who play outdoors with supervision of adults during the period of 2005 to 2013 in Norway [45]. A drawback of our survey is that it does not provide information about indoor and organized activities children are doing in their leisure time. However, there is a strong indication that a replacement in children’s play has taken place during the last decades, from outdoor play to more indoor and organized activities [45], and the children prefer screen-based activities instead of being outdoors playing with friends. An advantage of outdoor independent play in nearby nature is that it provides equal opportunities for all children, independent of social class and cultural background, in contrast to organized activities that systematically prevent certain groups from participating [17]. Outdoor play was an important arena for meeting friends and developing social networks in childhood [17], but today there are many fewer opportunities for meeting friends outdoors. There is a lack of knowledge on the extent to which organized activities may be an important substitute for developing social networks [56].

We would like to highlight an important point we have noted in the research so far. The one-sided focus on individuality in modern childhood research has been criticized [57,58], e.g., personal benefits of visiting nature for physical and mental health, well-being, and personal satisfaction. What we risk overlooking are things that people have in common with each other, and not least with nature, e.g., taking care of our common environment. The criticism comes in this case from a socially oriented researcher, Louise Chawla, who emphasizes the existential importance of nature for humans—for our ability to feel connected with the environment and to develop a moral relationship with nature. Removing children’s connection to nature may lead to detrimental effects on future generations’ conservation orientation and how they care for nature [57,59].

### 5.2. Significant Increase in Constraints on Children’s Greenspace Use

Regarding differences in parents’ evaluations of the 19 constraint statements between 2013 and 2023, we found significantly higher agreement with 17 of the 19 statements. The only two statements that did not show significant increases were “I/we parents are concerned about traffic” and “School homework takes too much time”. The most important constraints (or lack of motivation) were that children prefer to be indoors, are too busy during their leisure time, prefer screen-based activities, and miss friends to play with. Our findings contribute to the current body of knowledge, as previous research has primarily focused on declining access to nature and reduced perceived attractiveness of natural environments as key barriers to children’s engagement with nature [8]. In contrast, less has been paid to how children’s and adolescents’ socio-ecological everyday settings—such as time pressure, indoor activities, social media, and institutionalized or organized activities—affect their natural engagement [12,30,56]. In the following sections, we first discuss these findings in relation to different categories of constraints (Figure 1), then discuss the identified socio-cultural differences, and finally reflect on the limitations of the study.

Our results support the hypothesis that societal changes and modern-day lifestyles are reducing children’s time and motivation for outdoor play. Sometimes there is a lack of nearby natural areas for play or fewer usable spaces for play [13], but in Norway with often short distances to nature that is suitable for children’s play [31], access to nature is considered by the parents to be a minor constraint. Parents also report that concerns about traffic and stranger danger are of minor importance in the Norwegian context. Similarly, they consider their children to have good motor skills, sufficient proficiency to be outdoors, and to be well-equipped for outdoor activity. Thus, even though parents perceive that children in Norway have good access to nature and that nature areas are safe, well-facilitated, and suitable for play, these conditions are of little consequence if the children lack time or motivation to play outdoors. Our findings indicate that children have less motivation to be outdoors. Part of the explanation for this is that they often have no friends to play with outside and depend on their parents to arrange social play opportunities [39]. What was once taken for granted—that children would naturally meet and play with peers outdoors—no longer applies today. As a result, many children prefer to stay indoors and engage in screen-based activities. Even though the parents seem to have a key role in inspiring and “pushing” the children to play outdoors [22,27], our results reveal that the parents prioritize schoolwork and organized activities over playing outdoors. Our data suggests that the children have become more institutionalized, and that both contact with nature and meeting friends today mainly take place through kindergarten, school, after-school care, and organized activities [45]. There is still limited knowledge about how contact with nature and peer interaction occur for the “institutionalized” and “domesticated” child.

Our data show that the constraints on playing outdoors with friends are closely linked to how we organize our lives in contemporary society. It is a busy everyday life for both children and parents, and playing outdoors is often given lower priority compared to other chores and leisure activities. If more outdoor play is to be achieved, a shift in attitudes among parents and children is needed—one in which outdoor play is given greater value and priority in everyday life. But how is this to be achieved? A promising strategy may be to look more closely at the extent and form of outdoor play in institutions and in organized activities. It has been shown, for example, that there is great potential for facilitating independent play in organized forms, giving children time and space to let independent play unfold [11,30]. A challenge is that schools and organized activities are often preoccupied with the fact that they “have to offer the children something”—leading to programs that are highly structured and organized by adults [30]. Instead, the leaders could support outdoor play by creating more open time and space, reducing the level of adult control, and allowing for child-initiated, independent play [10,11,30].

### 5.3. Demographic, Socio-Economic, and Social Factors Explaining Children’s Greenspace Use

Our data show that there are some important socio-cultural differences regarding constraints for contact with nature and outdoor play amongst youth. Important age differences have been identified in our survey: the older the child is, the harder it is to get in touch with nature and be outdoors. This is in line with other studies that identify a decline in the frequency of nature use by youth compared with early childhood [26,27,59]. Young people have more schoolwork, a stronger preference for being indoors, and a desire to be active on screens, rather than with friends outdoors. Our analyses suggest that it is mainly before the age of 12 that children have the best opportunities for nature contact and independent play outdoors. In the past, it was common for older children to be outdoors looking after the younger ones, and outdoor activities increased with age and skill development [17,49]. Today, the situation is the opposite: younger children seem to spend more time outdoors, often together with adults in the family [6] or in organized settings [30,60].

We found a few gender differences in outdoor play and its constraints; the only significant constraint was that the parents were more concerned about screen activity for boys than for girls. Our survey does not measure time use, so it may be that parents expect boys to be more active outdoors than girls and therefore express more concern about boys’ screen use. Other surveys that look at time use have come to the opposite conclusion: that girls use screens more frequently, while boys are more active outdoors [16,19,20].

Regarding the gender of the parents, women believed that there were greater constraints related to equipment, and they also believed that their children spent more time on screen activities than men. This may indicate a gender difference in parents’ attitudes toward screen use, as women rated it as a greater constraint than men did. We have not found any other studies that have examined this variable.

We found that children who live in cities have somewhat less access to nature, and both children and parents have a busier everyday life. Our data reveal that there are greater constraints to outdoor play in urban areas than in more sparsely populated areas. An urban Dutch study identified that children’s favorite places are often playgrounds in built environments, and perhaps an emerging hypothesis is that children in urban settings become accustomed to easy-to-use and entertaining playgrounds which do not challenge them to be creative with their surroundings (e.g., build a cabin), which is a hallmark of play in nature [61]. Today, 83% of the population in Norway lives in built-up areas, and urbanization has resulted in a busy lifestyle [31]. Access (proximity) to good and safe natural areas can thus influence children’s engagement with nature in the most urban areas of Norway. Such differences have been shown in other studies, where access to nature and safety are important explanations for not using neighborhoods [18,40,41]. It is especially younger children that their parents are worried about, in terms of traffic and stranger danger.

For the other socio-cultural variables, we found few differences. We found that those who were single parents had fewer social networks than those who had multiple parents. Since much depends on the parents organizing with other parents so that the children can play outdoors with others, this can be an important constraint for those who have single parents.

### 5.4. Strengths and Limitations of the Study

The material from 2013 has more respondents (n = 3168) than the 2023 sample (n = 433), and the 2013 data is more robust in identifying socio-cultural differences. However, we found similar socio-demographic patterns in the 2023 material as in 2013, with one exception related to the fact that parents’ income was a more important constraint in the 2013 material. Since there is limited knowledge about what constrains or limits children’s contact with nature and outdoor play, the fact that it is nationally representative of parents with school-age children makes this study particularly valuable. Our results show that socio-cultural variables mediate the constraints for children and youth in their engagement with nature, and regarding green justice, this is something that should be studied more in future research. This coincides with the broad socio-ecological framework, which includes both socio-demographic variables and characteristics of the physical landscape [28]. Such a holistic approach can better capture how the various variables interact and show the complexity and intersection between individual, social, and structural constraints in order to get children and youth better engaged with nature, regardless of class.

The COVID-19 pandemic, with restrictions on behavior in 2021, has had a major impact on children’s and youth’s outdoor activities—and not least indoor activities with screens (e.g., [20,21]). This is an event our study has not considered. Even though society had stabilized before the survey was carried out in 2023, there is reason to assume that the legacy effects of the pandemic influenced the observed patterns in our survey. When it comes to the measurement dimensions used in our study, we have taken the literature as a starting point to define the most important constraints. It is a difficult exercise to formulate valid questions, especially because the constraints are highly contextual and strongly linked to each other. However, the defined constraints in the survey help paint a broad picture about how we live our lives in contemporary society and the place of outdoor play in everyday settings. Hence, we have also included questions more related to motivation and lack of motivation than to constraints alone. Asking the parents about their own children’s opportunities can introduce bias, as responses may reflect parental desires rather than the actual situation [27]. We attempted to reduce such bias through careful question design; however, we cannot rule out the influence of social desirability in responses. The decision to focus on the oldest child in the family also affected our sample composition. Children aged 6–8 years were underrepresented compared to those aged 10–12 years. The sampling approach may have influenced other variables as well, although we have not found studies that evaluate the effects of such a sampling frame.

## Figures and Tables

**Table 1:** Selected parameters used in the analyses of children’s nature use and constraints from survey questionnaires in 2013 and 2023.

Survey Details	Response Options
A. Questions about children’s outdoor use in different neighborhood settings	
What do your children do outdoors in the nearby environment during leisure time, and how often?	[dropdown, 5 categories:] - More often than weekly - Once a week - Three times a month - 1–2 times a month - More rarely
B. Demographic characteristics	
Parent: Age and gender, Income, Education, Single parent, Ethnicity, Postal code, Family structure, Number of children, Rural vs. urban living	[dropdown, age—continues] [dropdown, gender] [dropdown, income, 16 categories] [dropdown, education, 6 categories] [dropdown, sole parent, yes/no] [dropdown, ethnicity, 7 categories] [dropdown, family structure, 3 categories] [dropdown, number of children, specify] [dropdown, rural vs. urban living, 4 categories]
Child: Age and gender Rural vs. urban living, Postal code	[dropdown, age—continues] [dropdown, gender] [dropdown, urban-rural, 4 categories] [dropdown, postal code, specify]
C. Constraints on nature use	
To what extent do you agree or disagree that the following statements represent a hindrance for the child to visit nature or green spaces?	[dropdown, Likert scale 1–5, 1 = completely disagree, 5 = completely agree, 19 statements] - Distance to nature and other green areas is too far - The child is too busy with leisure time (organized sports and leisure activities) - We parents are concerned about traffic - There is too much bad weather - School homework takes too much time - Too high expenses to reach attractive nature and green areas - Too much demand for equipment, clothes, shoes, etc. - The child has poor motor skills - The child prefers being indoors - The nature and green areas are poorly facilitated - The child spends so much time on data and other screens that being outside is downgraded - The child does not want to play outdoors in nature - The child lacks friends who want and have time to visit nature and green areas - We parents find it unsafe in nature and green areas - We parents lack a social network that could support outdoor activities with the child - We parents prioritize playing and other activities indoors over being outside - We parents have a time schedule filled with work, activities, sports, and other things, and motivating the child to be outside is downgraded - We parents find schoolwork more important than motivating the child to be outside in nature and other green areas - We parents find participation in sports and other leisure activities more important than motivating the child to be outside in nature and other green areas

**Table 2:** Differences in survey data from 2013 and 2023 regarding parents’ experiences of constraints on children’s (6–12 years) nature use were analyzed using Welch’s *t*-test. Responses to nineteen constraint statements were recorded on a 5-point Likert-type scale, ranging from ‘completely disagree’ (score 1) to ‘completely agree’ (score 5). Significant differences between the 2013 and 2023 samples are marked in green. The five most important constraints are marked in orange for the 2013 sample and in blue for the 2023 sample.

To What Extent Do You Agree or Disagree That the Following Statements Are a Hindrance for the Child to Visit Nature or Green Spaces?	Year	N	Mean	Std. Deviation	Std. Error Mean	Test Statistics Welch’s *t*-Test
The distance to nature and other green areas is too far	2013	3162	1.74	1.153	0.020	t_3325_ = −6.903; *p* < 0.001; 2023 > 2013
	2023	426	2.18	1.356	0.066	
The child is too busy with leisure time (organized sports and leisure activities)	2013	3163	2.56	1.212	0.022	t_3325_ = −8.545; *p* < 0.001; 2023 > 2013
	2023	426	3.04	1.236	0.060	
We parents are concerned about traffic	2013	3166	2.51	1.312	0.023	t_3325_ = −0.520; *p* = 0.603; 2023 ≈ 2013
	2023	427	2.68	1.302	0.063	
There is too much bad weather	2013	3155	2.03	1.123	0.020	t_3325_ = −5.199; *p* < 0.001; 2023 > 2013
	2023	427	2.35	1.227	0.059	
School homework takes too much time	2013	3166	2.53	1.135	0.020	t_3325_ = −0.620; *p* = 0.535; 2023 ≈ 2013
	2023	427	2.49	1.216	0.059	
The expenses are too high to reach attractive nature and green areas	2013	3159	1.34	0.743	0.013	t_3325_ = −7.508; *p* < 0.001; 2023 > 2013
	2023	427	1.61	0.891	0.043	
There is too high demand for equipment, clothes, shoes, etc.	2013	3172	1.51	0.860	0.015	t_3325_ = −7.508; *p* < 0.001; 2023 > 2013
	2023	427	1.78	0.952	0.046	
The child has poor motor skills	2013	3170	1.24	0.701	0.012	t_3325_ = −5.883; *p* < 0.001; 2023 > 2013
	2023	427	1.45	0.898	0.043	
The child prefers being indoors	2013	3162	2.48	1.226	0.022	t_3325_ = −10.554; *p* < 0.001; 2023 > 2013
	2023	427	3.07	1.279	0.062	
The nature and green areas are poorly facilitated	2013	3139	1.58	0.920	0.016	t_3325_ = −4.571; *p* < 0.001; 2023 > 2013
	2023	427	1.78	1.057	0.051	
The child uses so much time on data and other screens that being outside is downgraded	2013	3166	2.34	1.221	0.022	t_3325_ = −9.030; *p* < 0.001; 2023 > 2013
	2023	426	2.80	1.339	0.065	
The child does not want to play outdoors in nature	2013	3159	2.08	1.107	0.020	t_3325_ = −10.962; *p* < 0.001; 2023 > 2013
	2023	427	2.61	1.192	0.058	
The child lacks friends who want and have time to visit nature and green areas	2013	3141	2.21	1.166	0.021	t_3325_ = −10.590; *p* < 0.001; 2023 > 2013
	2023	426	2.77	1.259	0.061	
We parents find it unsafe in nature and in green areas	2013	3159	1.45	0.858	0.015	t_3325_ = −4.224; *p* < 0.001; 2023 > 2013
	2023	427	1.69	1.002	0.048	
We parents lack a social network that could increase activity with the child outdoors	2013	3154	1.85	1.138	0.020	t_3325_ = −7.602; *p* < 0.001; 2023 > 2013
	2023	427	2.31	1.263	0.061	
We parents prioritize playing and other activities indoors above being outside	2013	3159	1.93	1.029	0.018	t_3325_ = −6.619; *p* < 0.001; 2023 > 2013
	2023	427	2.28	1.123	0.054	
We parents have a time schedule filled up with jobs, activities, sports, and other things, and motivating the child to be outside is downgraded	2013	3162	2.22	1.108	0.020	t_3325_ = −5.713; *p* < 0.001; 2023 > 2013
	2023	427	2.54	1.174	0.057	
We parents find schoolwork more important than motivating the child to be outside in nature and other green areas	2013	3156	2.28	1.097	0.020	t_3325_ = −3.777; *p* < 0.001; 2023 > 2013
	2023	426	2.48	1.133	0.055	
We parents find participation in sports and other leisure activities more important than motivating the child to be outside in nature and other green areas	2013	3146	2.18	1.081	0.019	t_3325_ = −2.797; *p* < 0.001; 2023 > 2013
	2023	427	2.34	1.020	0.049	

## Data Availability

The original contributions presented in this study are included in the article and Supplementary Materials. Further inquiries can be directed to the corresponding author.

## Supplementary Materials

The following supporting information can be downloaded at https://www.mdpi.com/article/10.3390/ijerph22071067/s1. Table S1: Associations between constraints for play and stay in nature, and demographic, socio-economic, and social factors in the 2013 survey (n = 3168); Table S2: Associations between constraints for play and stay in nature, and demographic, and social factors in the 2023 survey (n = 433).
