# Supplementary material for: Children’s Nature Use and Related Constraints: Nationwide Parental Surveys from Norway in 2013 and 2023
Source: Int J Environ Res Public Health. 2025 Jul 3;22(7):1067. doi: 10.3390/ijerph22071067 (PMC12294496; doi:10.3390/ijerph22071067)
Supplement: Supplementary file 1 [file ijerph-22-01067-s001.zip › ijerph-3669687-supplementary.pdf]

Table S1. *Associations between constraints for play and stay in nature, and demographic, socio-economic and social factors in the 2013 survey (n=3168).*

To what extent do you agree or disagree that the following statements are a hindrance for the child to visit nature or green spaces?	Child's gender (Boy/Girl)	Child's age (6-9 years/10-12 years)	Parent's income (<800 000 NOK/>800 000 NOK)	Parent's education level (Low/High)	Rural vs. urban living	Family structure (one home/two homes)	Sole parent (yes/no)	Country of origin (Norway/outside Norway)	Number of children? (one children/several children)	Parents gender (Women/men)	Parents age (25-39 years/40-72 years)
Distance to nature and other green areas is too far	-0,006	-0,028	0,030	,040*	,153**	0,014	,004	,015	-,037*	-,044*	,000
The child is too busy in leisure time (organized sports and leisure activities)	0,008	,111**	,113**	,081**	,143**	-0,002	,004	,014	,008	-,075**	,039*
I/we parents are concerned about traffic	0,015	-,150**	0,008	0,024	,037*	-,045*	,030	-,012	-,050**	-,066**	-,010
There is too much bad weather	0,005	0,003	-0,021	-,035*	,062**	0,026	-,013	-,007	-,013	-,030	-,030
School homework	-,038*	,100**	-,054**	-,057**	,059**	,046*	-,030	-,007	-,009	,011	-,029

takes too much time											
Too high expenses to reach attractive nature and green areas	-0,014	0,023	-,074**	-,068**	,045*	,050**	-,046*	,033	-,015	-,057**	,000
Too high demand for equipment, cloths, shoes etc.	0,002	0,027	-,086**	-,082**	,063**	,083**	-,017	-,005	-,002	-,118**	-,017
The child has poor motor skill	-,053**	0,018	-0,035	-,066**	0,012	,061**	,020	-,006	-,033	-,073**	-,016
The child prefers being indoors	-,075**	,116**	-0,006	,050**	,064**	,071**	-,001	-,011	-,046**	-,059**	-,009
The nature and green areas are poorly facilitated	-0,002	0,006	-0,011	-,038*	,081**	,047**	-,007	,034	-,043*	-,091**	,008
The child uses so much time on data and other screens that to be outside is downgraded	-,145**	,262**	-0,016	-0,004	0,033	,057**	-,008	,006	-,036*	-,134**	,104**
The child does not want to play outdoors in nature	-,068**	,172**	-0,020	0,007	,066**	,068**	-,042*	-,001	-,059**	-,039*	,052**

The child lacks friends who want and have time to visit nature and green areas	-,056**	,119**	-0,011	-0,006	,055**	,045*	-,017	,001	-,072**	-,049**	,030
I/we parents find it unsafe in nature and green areas	0,025	0,007	-0,016	-0,023	,073**	-0,006	-,043*	-,007	-,038*	-,024	,018
I/we parents lack social network that could increase activity with the child outside	-0,031	-0,003	-,090**	-,047**	,064**	,098**	-,121**	,018	-,080**	-,086**	-,019
I/we parents prioritize playing and other activities indoors above being outside	0,012	-0,004	-0,033	0,005	,089**	,053**	-,034	,006	,025	-,038*	-,047**
I/we parents have a time schedule filled up with job, activities, sports, and other things and to motivate the child to be	-0,006	,053**	0,002	0,033	,100**	,043*	-,010	,003	,030	-,076**	,020

outside is downgraded											
I/we parents find schoolwork more important than motivating the child to be outside in nature and other green areas	-0,012	,085**	0,024	-0,021	,074**	0,012	-,045*	-,005	-,022	-,089**	,038*
I/we parents find participation in sports and other leisure activities more important than motivating the child to be outside in nature and other green areas	-0,009	,086**	,067**	0,001	,099**	0,009	-,007	,003	-,009	-,103**	,043*

**p < 0.01, *p < 0.05

Table S2. *Associations between constraints for play and stay in nature, and demographic, and social factors in the 2023 survey (n=433).*

To what extent do you agree or disagree that the following statements are a hindrance for the child to visit nature or green spaces?	Child's gender (Boy/Girl)	Child's age (6-8 years/9-12 years)	Parent's income (<800 000 NOK/>800 000 NOK)	Parent's education level (Low/High)	Rural vs. urban living	Family structure (one home/two homes)	Sole parent (yes/no)	Country of origin (Norway/outside Norway)	Number of children? (one children/several children)	Parents gender (Women/men)	Parents age (25-39 years/40-72 years)
Distance to nature and other green areas is too far	0,006	-0,038	0,094	0,033	-,110*	-0,009	0,030	-0,002	0,063	-0,056	-,108*
The child is too busy in leisure time (organized sports and leisure activities)	0,087	0,035	,147**	0,046	-0,089	-0,006	0,033	0,019	,123*	0,006	-0,039
I/we parents are concerned about traffic	-0,061	-,234**	0,059	0,003	-,099*	-0,035	0,017	0,088	0,023	-0,003	-,127**
There is too much bad weather	0,050	-0,047	-0,076	-,097*	-0,022	-0,022	-0,014	-0,030	0,076	-0,036	-,143**
School homework takes too much time	-0,052	-0,076	-0,090	-,142**	0,072	-0,010	-0,031	0,002	0,069	-0,070	-,119*
Too high expenses to	-0,033	-0,025	0,034	0,046	-0,012	0,036	-0,001	,100*	0,025	-0,052	-,122*

reach attractive nature and green areas											
Too high demand for equipment, cloths, shoes etc.	-0,067	0,012	-0,039	-0,035	-0,010	0,019	-,111*	0,034	-0,011	-0,066	-,121*
The child has poor motor skill	-0,029	-0,006	-0,064	-0,045	0,012	0,085	-0,074	-0,008	-0,026	0,024	-,151**
The child prefers being indoors	-0,082	0,093	-,108*	-0,086	-0,028	0,012	-,095*	-0,004	-0,006	0,087	-0,047
The nature and green areas are poorly facilitated	-0,036	0,023	-0,040	-0,066	-0,007	0,018	-,162**	0,041	-0,002	0,014	-0,084
The child uses so much time on data and other screens that to be outside is downgraded	-0,077	,176**	-,155**	-0,085	-0,027	0,026	-0,083	-0,020	-0,004	0,042	0,006
The child does not want to play outdoors in nature	-0,003	,153**	-,160**	-0,086	-0,020	0,067	-0,091	-0,013	-0,088	0,003	0,029
The child lacks friends who want and have time to visit	0,001	,141**	-0,081	0,035	-0,030	0,076	-0,041	0,027	-,119*	-0,005	0,052

nature and green areas											
I/we parents find it unsafe in nature and green areas	0,007	-0,063	-0,067	-0,085	-0,011	0,033	-,175**	0,005	0,045	-0,037	-,130**
I/we parents lack social network that could increase activity with the child outside	0,022	-0,026	-,131*	-,103*	-0,001	0,026	-,154**	0,010	-0,067	-0,055	-0,041
I/we parents prioritize playing and other activities indoors above being outside	-0,035	-0,016	-,134**	-,111*	-0,056	0,004	-,124**	-0,076	-0,055	0,025	-,110*
I/we parents have a time schedule filled up with job, activities, sports, and other things and to motivate the child to be outside is downgraded	0,013	0,013	-0,021	-0,017	-0,076	0,010	-0,066	0,013	0,090	0,004	-0,041
I/we parents find school work more important	-0,035	0,014	0,034	0,003	-,114*	0,039	-0,084	-0,047	-0,025	,161**	-0,017

than motivating the child to be outside in nature and other green areas											
I/we parents find participation in sports and other leisure activities more important than motivating the child to be outside in nature and other green areas	0,036	0,032	-0,025	-,105*	-0,023	0,054	-0,073	-0,060	0,003	,099*	-0,021

**p < 0.01, *p < 0.05
